# Beyond Moments: Extending the Maximum Entropy Principle to Feature Distribution Constraints

**DOI:** 10.3390/e20090650

**Published:** 2018-08-30

**Authors:** Paul M. Baggenstoss

**Affiliations:** Fraunhofer FKIE, Wachtberg 53343, Germany; p.m.baggenstoss@ieee.org; Tel.: +49-228-9435-150

**Keywords:** maximum entropy principle, PDF projection, statistical inference

## Abstract

The maximum entropy principle introduced by Jaynes proposes that a data distribution should maximize the entropy subject to constraints imposed by the available knowledge. Jaynes provided a solution for the case when constraints were imposed on the expected value of a set of scalar functions of the data. These expected values are typically moments of the distribution. This paper describes how the method of maximum entropy PDF projection can be used to generalize the maximum entropy principle to constraints on the joint distribution of this set of functions.

## 1. Introduction

### 1.1. Jaynes’ Maximum Entropy Principle

The estimation of probability density functions (PDF) is the cornerstone of classical decision theory as applied to real-world problems. The maximum entropy principle of Jaynes [1] proposes that the PDF should have *maximum entropy* subject to constraints imposed by the knowledge one has about the density. Let x be a set of *N* random variables $x=[x_{1},x_{2}\ldots x_{N}]$. The entropy of the distribution p(x) is given by

(1) $$H\left\{p\left(x\right)\right\}=-\int_{x}p\left(x\right)\log\left(p\left(x\right)\right)dx.$$

Jaynes worked out the case when the knowledge about p(x) consists of the expected value of a set of *K* measurements. More precisely, he considered the *K* scalar functions $\phi_{1}\left(x\right),\phi_{2}\left(x\right)\ldots \phi_{K}\left(x\right)$ and constrained the expected values:

(2) $$\int_{x}\phi_{k}\left(x\right)\;p\left(x\right)\;dx=d_{k},\;\;\;1\leq k\leq K.$$

If $\phi_{k}\left(x\right)=\sum_{i=1}^{N}x_{i}^{k},$ then (2) are moment constraints.

The distribution maximizing (1) subject to (2) is:

$$p\left(x\right)=e^{-[\lambda_{0}+\lambda_{1}\phi_{1}\left(x\right)+\lambda_{2}\phi_{2}\left(x\right)+\ldots \lambda_{K}\phi_{K}\left(x\right)]},$$

where $\lambda_{0}$ is the log of the partition function:

$$Z\left(\lambda_{1},\lambda_{2}\ldots \lambda_{K}\right)=\int_{x}e^{-[\lambda_{1}\phi_{1}\left(x\right)+\lambda_{2}\phi_{2}\left(x\right)+\ldots \lambda_{K}\phi_{K}\left(x\right)]}.$$

The constants $\lambda_{k}$ are determined by solving

$$d_{k}=\frac{\partial }{\partial \lambda_{k}}\log Z,\;\;\;1\leq k\leq K.$$

### 1.2. Feature Distribution Constraints

Jaynes’ results had initial applications in statistical mechanics and thermodynamics [2], and have found more applications in a wide range of disciplines [2,3,4,5]. However, we would like to extend the results by replacing constraints (2) with constraints that are more meaningful in real-world inference problems. Instead of knowing just the average values of $\phi_{k}\left(x\right)$, suppose we knew the *joint distribution* of $z=\Phi \left(x\right)=[\phi_{1}\left(x\right),\phi_{2}\left(x\right),\ldots \phi_{K}\left(x\right)]$, denoted by $p_{z}\left(z\right)$. This carries more information than the average values of each measurement $\phi_{k}\left(x\right)$. Because the number of parameters *K* is small compared with the dimension of x, it is feasible to estimate $p_{z}\left(z\right)$ from a set of training samples using kernel-based PDF estimation methods, for example. This constraint is more general and can be adapted to produce something similar to Jaynes’ constraints (2) if the marginal measurement distributions are assumed independent, and Gaussian with mean $d_{k}$. This has immediate applications in a wide range of fields, for example in speech analysis and recognition where z could be MEL frequency cepstrum coefficients (MFCC) [6] extracted from the time-series data x, or in neural networks, where z could be the output of a network.

Note that the distribution $p_{z}\left(z\right)$ can be obtained from p(x) by marginalization:

(3) $$p_{z}\left(z\right)=\int_{x\in M(z)}\;p\left(x\right)\;dx,$$

where the integral is carried out on the *level set* or manifold given by

(4) $$M\left(z\right)=\{x:\phi_{1}\left(x\right)=z_{1},\phi_{2}\left(x\right)=z_{2},\ldots \phi_{K}\left(x\right)=z_{K}\}.$$

The constraint problem can then be re-stated as follows:

**Problem** **1.** *Given a known distribution $p_{z}\left(z\right)$, maximize the entropy of p(x) subject to*

(5) $$\int_{x\in M(z)}\;p\left(x\right)\;dx=p_{z}\left(z\right),\;\;\;\;\forall z.$$

The solution to this problem is called maximum entropy PDF projection [7,8,9].

### 1.3. Significance

The main significance of maximum entropy PDF projection is the de facto creation of a statistical model through the extraction of features. Once a feature extraction z=Φ(x) has been identified, and it meets some mild requirements given below, a statistical model has been determined. This has a number of advantages, not the least of which is that the “art” of extracting features, i.e., signal processing, is well established, and many good methods exist to extract meaningful information from data. For example, the extraction MFCC features for processing speech signals has been developed to approximate human hearing [6], and, therefore, with maximum entropy PDF projection, should lead to statistical data models which share some qualities with human perception. Before maximum entropy PDF projection, comparing feature extraction methods had to be done based on secondary factors such as classification results. Maximum entropy PDF projection allows a feature extraction method to be evaluated based its corresponding statistical model.

**Figure 1:**
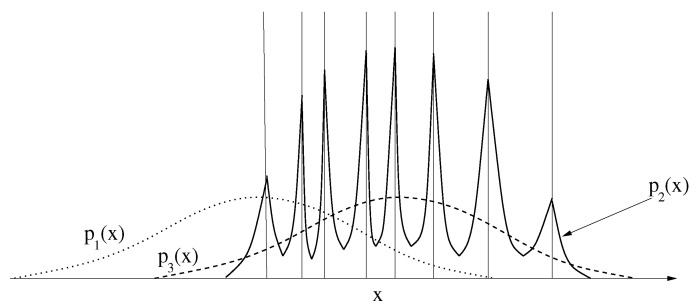
Comparison of entropy *Q* and average log-likelihood *L* for three distributions. The vertical lines are the locations of training samples.

The use of the maximum entropy principle assures the fairest means of comparing two statistical models derived from competing feature extraction methods. In most real-world applications, we cannot know p(x), and must be satisfied with estimating it from some training data. Suppose that we have a set of *K* training samples $x_{1},\;x_{2},\;\ldots ,x_{K}$, and have a number of proposed PDFs computed using (6) for various feature transformations $z_{i}=\Phi_{i}\left(x\right)$. Let these projected PDFs be denoted by $p_{i}\left(x\right)$. We would like to determine which projected PDF (i.e., which feature vector) provides a “better” fit to the data. One approach would be to compare the PDFs based on the average log-likelihood $L_{i}=\frac{1}{K}\sum_{n=1}^{K}\log p_{i}\left(x_{n}\right),$ choosing the feature transformation that results in the largest value. However, likelihood comparison by itself is misleading, so one must also consider the entropy of the distribution, $Q_{i}=-\int_{x}\left\{\log p_{i}\left(x\right)\right\}\;p_{i}\left(x\right)dx,$ which is the negative of the theoretical value of $L_{i}$. Distributions that spread the probability mass over a wider area have higher entropy since the average value of logp(x) is lower. The two concepts of *Q* and *L* are compared in Figure 1 in which we show three competing distributions: $p_{1}\left(x\right)$, $p_{2}\left(x\right)$, and $p_{3}\left(x\right)$. The vertical lines represent the location of the *K* training samples. If $L_{i}$ is the average value of $\log p_{i}\left(x\right)$ at the training sample locations, then clearly $L_{1}\ll L_{3}\ll L_{2}.$ However, choosing $p_{2}\left(x\right)$ is very risky because it is over-adapted to the training samples. Clearly, $p_{2}\left(x\right)$ has lower entropy since most of the probability mass is at places with higher likelihood. Therefore, it has achieved higher *L* at the cost of lower *Q*, a suspicious situation. On the other hand, $Q_{1}=Q_{3}$, but $L_{3}>L_{1}$. Therefore, $p_{3}\left(x\right)$ has achieved higher *L* than $p_{1}\left(x\right)$ without suffering lower *Q*, so choosing $p_{3}\left(x\right)$ over $p_{1}\left(x\right)$ is not risky. If we always choose among models that have maximum possible entropy for the given choice of features, we are likely to obtain better features and better generative models.

## 2. Main Results

### 2.1. MaxEnt PDF Projection

The solution to Problem 1 is based on PDF projection [10]. In PDF projection, one is given a feature distribution $p_{z}\left(z\right)$ and constructs a PDF as follows:

(6) $$p_{p}\left(x;p_{0},\Phi ,p_{z}\right)=\frac{p_{0}\left(x\right)}{p_{0,z}\left(z\right)}\;p_{z}\left(\Phi \left(x\right)\right),$$

where $p_{0}\left(x\right)$ is a reference distribution meeting some mild constraints [10], and $p_{0,z}\left(z\right)$ is the corresponding distribution imposed by $p_{0}\left(x\right)$ on the measurements z, i.e., $p_{0,z}\left(z\right)$ is Equation (3) applied to $p_{0}\left(x\right)$. It can be shown that

- Equation (6) is a PDF, so $\int_{x}p_{p}\left(x;p_{0},\Phi ,p_{z}\right)dx=1.$
- $p_{p}\left(x;p_{0},\Phi ,p_{z}\right)$ meets (5), so it is consistent with $p_{z}\left(z\right)$.
- All distributions meeting (5) can be written in the form (6) for some $p_{0}\left(x\right)$.

The last item in the list indicates that, to solve Problem 1, it is only necessary to select the reference distribution $p_{0}\left(x\right)$ for *maximum entropy* (MaxEnt).

To understand the solution to this problem, it is useful to consider the sampling procedure for (6). To sample from distribution (6), one draws a sample $z^{*}$ from PDF $p_{z}\left(z\right)$; then, x is drawn from the set $M(z^{*})$, defined in (4). Note, however, that to conform to (6), it is necessary to draw sample x from $M(z^{*})$ with probability proportional to the value of $p_{0}\left(x\right)$. The distribution of x on the manifold $M(z^{*})$ may be thought of the conditional distribution $p(x|z^{*})$, and it is proportional to $p_{0}\left(x\right)$. It is in fact

(7) $$p\left(x|z^{*}\right)=\frac{p_{0}\left(x\right)}{p_{0,z}\left(z\right)}.$$

It can be verified that (7) integrates to 1 on the manifold $M(z^{*})$. The entropy of (6) can be decomposed as the entropy of $p_{z}\left(z\right)$ plus the expected value of the entropy of the p(x|z) (see Equation (8) in [8]):

$$H\left\{p_{p}\left(x;p_{0},\Phi ,p_{z}\right)\right\}=H\left\{p_{z}\left(z\right)\right\}\;+\;\int_{z}H\left\{p\left(x|z\right)\right\}\;p_{z}\left(z\right)\;dz.$$

Maximizing this quantity seems daunting, but there is one condition under which H{p(x|z)} has the maximum entropy for all z, and that is when p(x|z) is the *uniform* distribution for all z. This, in turn, is achieved when $p_{0}\left(x\right)$ has a constant value on any manifold M(z).

This process of selecting $p_{0}\left(x\right)$ for maximum entropy is called maximum entropy PDF projection [8,9]. The maximizing reference distribution is written $p_{0}^{*}=\arg\max_{p_{0}}H\left\{p_{p}\left(x;p_{0},\Phi ,p_{z}\right)\right\},$ and the MaxEnt distribution is written

(8) $$p_{p}^{*}\left(x;\Phi ,p_{z}\right)=\frac{p_{0}^{*}\left(x\right)}{p_{0,z}^{*}\left(z\right)}\;p_{z}\left(\Phi \left(x\right)\right),$$

which is the unique distribution that solves Problem 1.

In order that it is possible to select $p_{0}\left(x\right)$ for MaxEnt, the feature transformation Φ(x) must be such that the uniform distribution can be defined on M(z) for any z. Thus, M(z) must be bounded and integrable. This condition is easily met if the feature z contains information about the size of x so that when z is fixed to a finite value, the x has a fixed norm. To say this formally, let there exist a function f(z) such that f(Φ(x))=∥x∥ for some valid norm ∥x∥ on the range of x.

Once this condition is met, then $p_{0}^{*}\left(x\right)$ is any distribution that is constant on any level set M(z). This happens if there exists a function *c* such that

$$p_{0}^{*}\left(x\right)=c\left(\Phi \left(x\right)\right).$$

Interestingly, any $p_{0}^{*}\left(x\right)$ meeting these constraints results in the same distribution (6) [8]. This means that, although $p_{0}^{*}\left(x\right)$ is not unique, $p_{p}^{*}\left(x;\Phi ,p_{z}\right)$ is *unique*—it *must* be unique if it is the maximum entropy PDF.

The above conditions can be easily met by inserting an *energy statistic* into the feature set Φ(x), and defining a reference distribution that depends on x only through this energy statistic. The energy statistic is a scalar statistic from which it is possible to compute a valid norm on the range of x, denoted by X. In summary, the simplest way to solve for the MaxEnt projected PDF given the range of x, denoted by X, involves these three steps:

1. Identify a norm ∥x∥ valid in X A norm ∥x∥ must meet the properties of scalability ∥ax∥=|a|∥x∥, triangle inequality ∥x+y∥≤∥x∥+∥y∥, and ∥0∥=0.
2. Identify a scalar statistic (energy statistic) t(x) from which it is possible to compute ∥x∥:
  ∥x∥=f(t(x)).
3. Use a reference hypothesis depending only on t(x).

The above will be demonstrated for three cases of X in Section 3.1, Section 3.2 and Section 3.3.

The data generation process for MaxEnt PDF projection, corresponding to distribution (8) does not depend on X and is the following:

1. From the known distribution $p_{z}\left(z\right)$, draw a sample denoted by $z^{*}=\left[z_{1}^{*},z_{2}^{*}\ldots z_{K}^{*}\right]$.
2. Now identify the set of all samples x mapping to $z^{*}$, denoted by $M(z^{*}).$
3. Draw a sample x from this set, uniformly, so that no member of $M(z^{*})$ is more likely to be chosen than another.

The maximum entropy nature of the solution can be recognized in the uniform sampling on the level set $M(z^{*}).$ The last item above is called uniform manifold sampling (UMS) [9]. The data generation process for three cases of X are provided in Section 3.1, Section 3.2 and Section 3.3.

## 3. Examples

The implementation of MaxEnt PDF projection depends strongly on the range of the input data x, denoted by X. In this section, examples are provided for three important cases of X.

### 3.1. Unbounded Data $X=R^{N}$

Let x range everywhere in $R^{N}$. The 2-norm ${∥x∥}_{2}$ is valid in $R^{N}$ and can be computed from the total energy

$$t_{2}\left(x\right)=\sum_{n=1}^{N}x_{n}^{2}.$$

The Gaussian reference hypothesis can be written in terms of $t_{2}\left(x\right)$:

(9) $$p_{0}\left(x\right)=\prod_{i=1}^{N}\frac{1}{\sqrt{2\pi }}e^{-x_{i}^{2}/2}={\left(2\pi \right)}^{-N/2}\;e^{-t_{2}\left(x\right)/2},$$

so naturally $p_{0}\left(x\right)$ will have a constant value on any manifold M(z). Naturally, it is not necessary to include $t_{2}\left(x\right)$ explicitly in the feature set—it is only necessary that the 2-norm can be computed from z.

The distribution $p_{0,z}^{*}\left(z\right)$ can be determined in closed form for some feature transformations [11,12]. For others, the moment generating function can be written in closed form, which allows the saddle point approximation to be used to compute $p_{0,z}^{*}\left(z\right)$ [11]. More on this will be presented in Section 4.1.

An important case where a closed-form solution exists is the linear transformation combined with total energy:

$$z=[A^{\prime }x,\;x^{\prime }x].$$

This case is covered in detail in ([8], Section IV.C, p. 2821), and in ([9], Section III.B, p. 2459).

The following simple example demonstrates the main points of this case. Assume input data dimension N=3 and a feature transformation consisting of the sample mean and sample variance:

$$z=\left[\hat{\mu },\hat{v}\right],$$

where

$$\hat{\mu }=\frac{1}{N}\sum_{i=1}^{N}x_{i},\;\;\;\hat{v}=\frac{1}{N-1}\sum_{i=1}^{N}{\left(x_{i}-\hat{\mu }\right)}^{2}.$$

Note that $t_{2}\left(x\right)$ can be computed from $\left(\hat{\mu },\hat{v}\right)$,

$$t_{2}\left(x\right)=\left(N-1\right)\hat{v}+N{\hat{\mu }}^{2},$$

which satisfies the requirement that the 2-norm of x can be computed from z.

**Figure 2:**
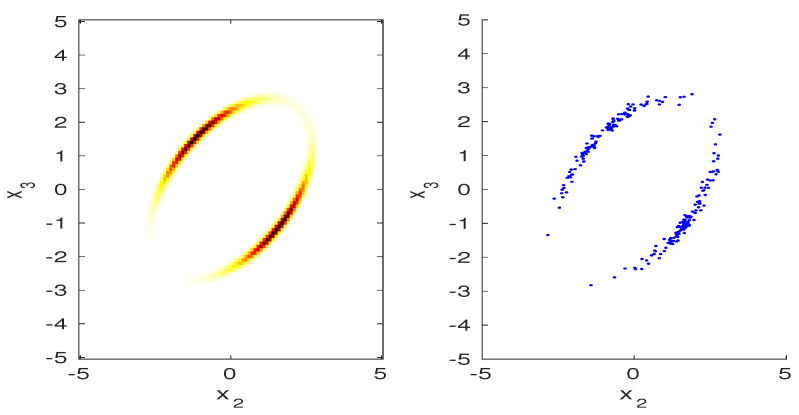
(**Left**) illustration of projected PDF for $\mu_{0}=0.15$, $v_{0}=0.3,$ $\mu_{1}=1.85$, $v_{1}=0.025$, on a slice of $x_{2}$, $x_{3}$ at $x_{1}=0$; (**Right**) samples drawn from the sampling procedure (see text).

Under the assumption that x is distributed according to the standard Normal distribution (9), $\hat{\mu }$ will have mean 0 and variance 1/N,

$$p_{0}\left(\mu \right)={\left(\frac{2\pi }{N}\right)}^{-1/2}\;e^{-N\mu^{2}/2},$$

and $\hat{v}$ will have the chi-square distribution with N−1 degrees of freedom and scaling $\frac{1}{N-1}$, which is given by

$$p_{0}\left(v\right)=\frac{k}{2^{k/2}}\;\Gamma^{-1}\left(\frac{k}{2}\right){\left[kv\right]}^{k/2-1}e^{-\frac{vk}{2}},$$

where k=N−1. Furthermore, $\hat{\mu }$ and $\hat{v}$ are statistically independent. Therefore, $p_{0,z}^{*}\left(z\right)=p_{0}\left(\mu \right)\cdot p_{0}\left(v\right).$ For the given feature distribution, we assume components of z are independent and Gaussian

$$p_{z}\left(z_{i}\right)={\left(2\pi v_{i}\right)}^{-1/2}e^{-{\left(z_{i}-\mu_{i}\right)}^{2}/\left(2v_{i}\right)}$$

with given mean $\mu_{i}$ and variance $v_{i}$, where $z_{0}=\hat{\mu },$ $z_{1}=\hat{v}$. The MaxEnt projected PDF, given by $p_{p}^{*}\left(x;\Phi ,p_{z}\right)=\frac{p_{0}^{*}\left(x\right)}{p_{0,z}^{*}\left(z\right)}\;p_{z}\left(z\right)$ is plotted on the left of Figure 2 for slice of $x_{2},x_{3}$ at $x_{1}=0.0$. The density values shown in the figure, summed over all three axes and properly scaled added to a value 0.9999999998, which validates with numerical integration that $p_{p}^{*}\left(x;\Phi ,p_{z}\right)$ is a density. Notice that the probability is concentrated on a circular region. This can be understood in terms of the sampling procedure given below.

To sample from $p_{p}^{*}\left(x;\Phi ,p_{z}\right)$, we first draw a sample of z from $p_{0,z}^{*}\left(z\right)$, denoted by $z^{*}$, which provides values for the sample mean value $\mu^{*}$ and variance $v^{*}$. Then, x must be drawn uniformly from the manifold $\left\{x:\hat{\mu }=\mu^{*},\;\;\;\hat{v}=v^{*}\right\},$ which are conditions on the sample mean and variance. This is easily accomplished if we note that the sample mean condition is met for any x of the form

(10) $$x={\left[1,1\ldots 1\right]}^{\prime }\mu^{*}+Bu,$$

where B is the N×(N−1) ortho-normal matrix spanning the space orthogonal to the vector ${\left[1,1\ldots 1\right]}^{\prime }.$ To meet the second (variance) condition, it is necessary that

$${∥u∥}^{2}=\left(N-1\right)v^{*}.$$

This condition defines a hypersphere in (N−1) dimensions, which explains the circular region in Figure 2. This hypersphere is sampled uniformly by drawing N−1 independent Gaussian random variables, denoted by u, then scaling u so that ${∥u∥}^{2}=\left(N-1\right)v^{*}.$ Then, x is constructed using (10). Samples drawn in this manner are shown on the right side of Figure 2. To agree with the left side of the figure, only samples with $|x_{1}|<0.01$ are plotted.

Please see the above-cited references for using general linear transformations.

### 3.2. Positive Data $X=P^{N}$

Let x have positive-valued elements, so x ranges in the positive quadrant of $R^{N}$, denoted by $P^{N}$. This holds whenever spectral or intensity data is processed. The appropriate norm in this space is the 1-norm

$$∥x∥=\frac{1}{N}\sum_{n=1}^{N}x_{n}.$$

To satisfy conditions for maximum entropy, it must be possible to compute the statistic $t_{1}\left(x\right)=\sum_{n=1}^{N}x_{n}$ from the features. The exponential reference hypothesis can be written in terms of $t_{1}\left(x\right)$:

(11) $$p_{0}\left(x\right)=\prod_{i=1}^{N}e^{-x_{i}}=e^{-t_{1}\left(x\right)},$$

so naturally $p_{0}\left(x\right)$ will have a constant value on any manifold M(z), and is the appropriate reference hypothesis for maximum entropy. The inclusion of $t_{1}\left(x\right)$ explicitly in the feature set is only one way to insure that M(z) is compact—it is only necessary that the 1-norm can be computed from z.

An important feature extraction is the linear transformation

$$z=A^{\prime }x.$$

Note that is necessary that statistic $t_{1}\left(x\right)$ can be computed from z, which can be accomplished, for example, to making the first column of A constant. This case is covered in detail in ([8], Section IV.B, p. 2820), and in ([9], Section IV, p. 2460). Sampling x is accomplished by drawing a sample $z^{*}$ from $p_{z}\left(z\right)$ and then drawing a sample x uniformly from the set $\{x:A^{\prime }x=z^{*}\}.$

The following simple example demonstrates the main theoretical concepts. We assume a data dimension of N=2 so that the distribution can be visualized as an image. The feature transformation is simply the sum of the samples:

$$z=T\left(x_{1},x_{2}\right)=x_{1}+x_{2}.$$

**Figure 3:**
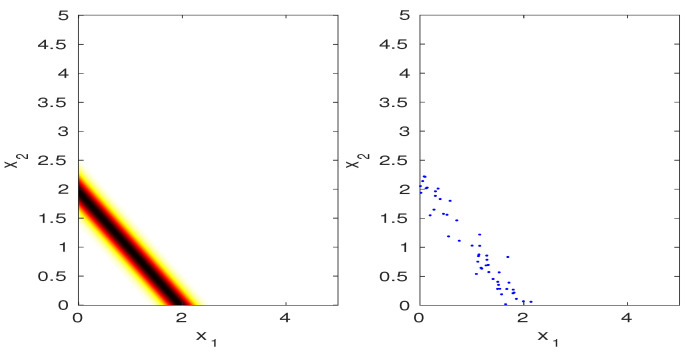
(**Left**) illustration of projected PDF for $\mu_{z}=2.0$, $v_{z}=0.04$; (**Right**) samples drawn from the sampling procedure (see text).

Under the exponential reference hypothesis, the feature distribution is chi-square with 2N degrees of freedom and scaling 1/2:

$$p_{0,z}^{*}\left(z\right)=\frac{2}{\Gamma (k/2)}\;2^{-k/2}\;{\left(2z\right)}^{\left(k/2-1\right)}e^{-z},$$

where k=2N. For the given feature distribution, we assume Gaussian

$$p_{z}\left(z\right)={\left(2\pi v_{z}\right)}^{-1/2}e^{-{\left(z-\mu_{z}\right)}^{2}/\left(2v_{z}\right)}$$

with a given mean $\mu_{z}$ and variance $v_{z}$. The MaxEnt projected PDF, given by $p_{p}^{*}\left(x;\Phi ,p_{z}\right)=\frac{p_{0}^{*}\left(x\right)}{p_{0,z}^{*}\left(z\right)}\;p_{z}\left(z\right)$ is plotted in Figure 3. The density values shown in the figure, when properly scaled, summed to a value 0.9998, which validates with numerical integration that $p_{p}^{*}\left(x;\Phi ,p_{z}\right)$ is a density. Note that the distribution is concentrated on the line $x_{1}+x_{2}=\mu_{z}=2$, and is flat on this line, as would be expected for maximum entropy. To sample from this distribution, we first draw a sample $z^{*}$ from $p_{z}\left(z\right)$ and then draw a sample x on the line given by $x_{1}+x_{2}=z^{*}$. This can be done by sampling $x_{1}$ uniformly in $\left[0,z^{*}\right]$, then letting $x_{2}=z^{*}-x_{1}.$ Samples drawn in this way are shown on the right side of Figure 3.

This example generalizes to higher dimension and to arbitrary linear transformations $z=A^{\prime }x$ for full-rank N×M matrix A. In this case, $p_{0,z}^{*}\left(z\right)$ is not chi-square, and in fact is not available in closed-form. However, the moment-generating function is available in closed-form so the saddle point approximation may be used (See Section IV.A, p. 2245 in [11]). Samples of x are drawn by drawing a sample $z^{*}$ from $p_{z}\left(z\right)$ and then sampling uniformly in the set $\left\{x:\;A^{\prime }x=z^{*}\right\}$. At high dimensions, this requires a form of Gibbs sampling called hit and run (see Section IV, p. 2460 in [9]).

### 3.3. Unit Hypercube, $X=U^{N}$

Let x have elements limited to $0\leq x_{i}\leq 1$. This case is common when working with neural networks. This is called the unit hypercube, denoted by $U^{N}$. The uniform reference hypothesis

(12) $$p_{0}\left(x\right)=1.$$

produces maximum entropy. No norm-producing energy statistic is needed. Naturally, $p_{0}\left(x\right)$ will have a constant value on any manifold M(z).

The following simple example demonstrates the main theoretical concepts. We assume a data dimension of N=2 so that the distribution can be visualized as an image. The feature transformation is simple the sum of the samples:

$$z=T\left(x_{1},x_{2}\right)=x_{1}+x_{2}.$$

For this case, the uniform distribution brings maximum entropy, $p_{0}^{*}\left(x\right)=1.$ Under the reference hypothesis, the feature distribution is Irwin-Hall, given by

p0,z*(z)=12(N−1)!∑k=0N(−1)kNk(z−k)N−1sign(z−k),

where sign(0)=0. For N=2, this is a triangular distribution

$$p_{0,z}^{*}\left(z\right)=\left\{z,\;\;0\leq z\leq 1;\;\;\;\;\;\;2-z,\;\;1\leq z\leq 2\right\}.$$

**Figure 4:**
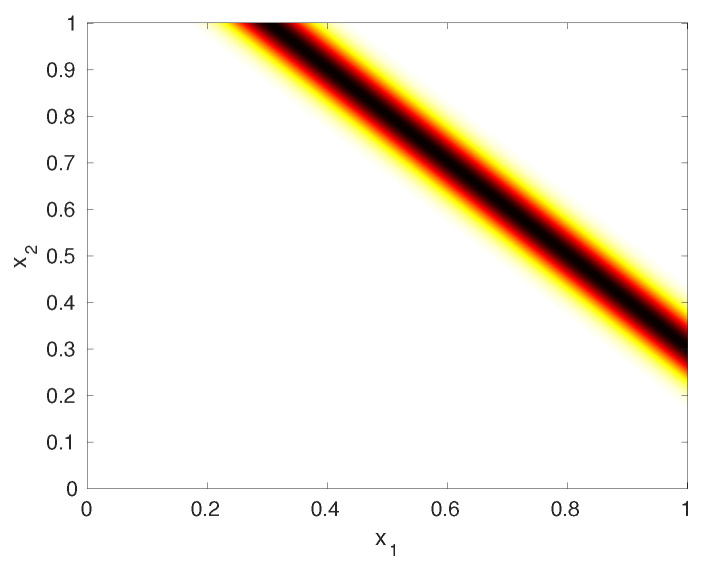
Illustration of projected PDF for $\mu_{z}=1.3$, $v_{z}=0.002$.

For the given feature distribution, we assume Gaussian

$$p_{z}\left(z\right)={\left(2\pi v_{z}\right)}^{-1/2}e^{-{\left(z-\mu_{z}\right)}^{2}/\left(2v_{z}\right)}$$

with a given mean $\mu_{z}$ and variance $v_{z}$. The MaxEnt projected PDF, given by $p_{p}^{*}\left(x;\Phi ,p_{z}\right)=\frac{p_{0}^{*}\left(x\right)}{p_{0,z}^{*}\left(z\right)}\;p_{z}\left(z\right)$ is plotted in Figure 4. The density values shown in the figure, when properly scaled, summed to a value 0.999, which validates with numerical integration that $p_{p}^{*}\left(x;\Phi ,p_{z}\right)$ is a density. Note that the distribution is concentrated on the line $x_{1}+x_{2}=\mu_{z}$, and is flat on this line, as would be expected for maximum entropy. To sample from this distribution, we first draw a sample $z^{*}$ from $p_{z}\left(z\right)$ and then draw a sample x on the line given by $x_{1}+x_{2}=z^{*}$. This can be done by finding where the line that intercepts the axes, and sampling uniformly in the interval between the intercepts. Note that this sampling differs from the previous example as a result of the upper bound at 1.

This example generalizes to higher dimension and to arbitrary linear transformations $z=A^{\prime }x$ for full-rank N×M matrix A. In this case, $p_{0,z}^{*}\left(z\right)$ is no longer Irwin-Hall and in fact is not available in closed-form. However, the moment-generating function is available in closed-form so the saddle point approximation may be used (see Appendix in [13]). Samples of x are drawn by drawing a sample $z^{*}$ from $p_{z}\left(z\right)$ and then sampling uniformly in the set $\left\{x:\;A^{\prime }x=z^{*}\right\}$. At high dimensions, this requires a form of Gibbs sampling called hit and run (see p. 2465 in [9]).

## 4. Advanced Concepts

### 4.1. Implementation Issues

Implementing (8) seems like a daunting numerical task, since $p_{0}^{*}\left(x\right)$ is some canonical distribution, for which a real data sample x normally lies in the far tails of both $p_{0}^{*}\left(x\right)$ and $p_{0,z}^{*}\left(z\right)$. However, if the distributions are known exactly, and are represented in the log domain, then the difference

(13) $$\log p_{0}^{*}\left(x\right)-\log p_{0,z}^{*}\left(z\right)$$

typically remains within very reasonable limits. In some cases, terms in $\log p_{0}^{*}\left(x\right)$ and $\log p_{0,z}^{*}\left(z\right)$ cancel, leaving (13) only weakly dependent on x (for example, see Section IV.A, p. 2820 in [8]).

Evaluating $\log p_{0}^{*}\left(x\right)$ is mostly trivial since it is normally a canonical distribution, such as Gaussian, exponential, or uniform. Calculating $\log p_{0,z}^{*}\left(z\right)$, however, remains the primary challenge in maximum entropy PDF projection. However, when evaluating $p_{0,z}^{*}\left(z\right)$ seems daunting, there are several ways to overcome the problem.

1. Saddle Point Approximation. If $p_{0,z}^{*}\left(z\right)$ is not available in closed form, the moment-generating function (MGF) might be tractable. This allows the saddle point approximation (SPA) to be used (see Section III in [11]). Note that the term “approximation” is misleading because the SPA approximates the shape of the MGF on a contour, not the absolute value, so the SPA expression for $\log p_{0,z}^{*}\left(z\right)$ remains very accurate, in the far tails, even when $p_{0,z}^{*}\left(z\right)$ itself cannot be evaluated in machine precision. Examples of this include general linear transformations of exponential and chi-squared random variables (see Section III.C and Section IV in [11]), general linear transformations of uniform random variables (Appendix in [13]), a set of linear-quadratic forms [14], and order statistics [15].
2. Floating reference hypothesis. There are conditions under which the MaxEnt reference hypothesis $p_{0}^{*}\left(x\right)$ is not unique, so it can depend on a parameter θ, so we write $p_{0}^{*}\left(x;\theta \right)$. An example is when the feature z contains the sample mean and sample variance (see example in Section 3.1). In this case, a Gaussian reference hypothesis $p_{0}^{*}\left(x;\theta \right)$ can be modified to have any mean and variance $\theta =[\mu_{0},\sigma_{0}^{2}]$, and can serve as the MaxEnt reference hypothesis with no change at all in the resulting projected PDF. In other words, (13) is independent of θ—this can be verified by cancelling terms. Therefore, there is no reason that θ cannot be made to track the data—that is, let $\mu_{0}=\hat{\mu }\left(x\right)$, $\sigma_{0}^{2}={\hat{\sigma }}^{2}\left(x\right)$. By doing this, $p_{0,z}^{*}\left(z\right)$ will track z, allowing simple approximations based on central limit theorem to be used to approximate $p_{0,z}^{*}\left(z\right)$.
3. Chain Rule. When $p_{0,z}^{*}\left(z\right)$ cannot be derived for a feature transformation, it may be possible to break the feature transformation into stages, where each stage can be easily analyzed. The next section is devoted to this.

### 4.2. Chain Rule

The primary numerical difficulty in implementing (8) is the calculation of $p_{0,z}^{*}\left(z\right)$. Solutions for many of the most useful feature transformations are available [9,11,12,13]. However, in many real-world applications, such as neural networks, the feature transformations cannot be easily written in a compact form $z=[\phi_{1}\left(x\right),\phi_{2}\left(x\right),\ldots \phi_{K}\left(x\right)]$. Instead, they consist of multi-stage transformations, for example, $y=T_{1}\left(x\right)$, $w=T_{2}\left(y\right)$, and $z=T_{3}\left(w\right).$ The individual stages $T_{m}\left(x\right)$ could be the layers of a neural network. In this case, it is best to apply (8) recursively to each stage. This means that the distribution of the first stage features p(y) is written using (6) with y taking the role of input data, and so forth. This results in the chain-rule form:

(14) $$p\left(x\right)=\left[\frac{p_{0,x}^{*}\left(x\right)}{p_{0,x}^{*}\left(y\right)}\right]\left[\frac{p_{0,y}^{*}\left(y\right)}{p_{0,y}^{*}\left(w\right)}\right]\left[\frac{p_{0,w}^{*}\left(w\right)}{p_{0,w}^{*}\left(z\right)}\right]\;p\left(z\right),$$

where $p_{0,x}^{*}\left(x\right)$, $p_{0,y}^{*}\left(y\right)$, $p_{0,w}^{*}\left(w\right)$ are canonical reference hypotheses used at each stage, for example (9), (11), and (12), depending on the range of x, y, and w, respectively.

To understand the importance of the chain-rule, consider how we would compute (6) without the chain rule. Let T(x) be the combined transformation

$$T\left(x\right)=T_{3}\left(T_{2}\left(T_{1}\left(x\right)\right)\right)$$

and let $p_{0}^{*}\left(x\right)$ be one of the canonical reference distributions. Consider the difficulty in deriving $p_{0,z}^{*}\left(z\right)$. At each stage, the distribution of the output feature becomes more and more intractable, and trying to estimate $p_{0,z}^{*}\left(z\right)$ is futile because generally a canonical reference distribution is completely unrealistic as PDF for real data. Furthermore, $p_{0,z}^{*}\left(z\right)$ is more often than not evaluated in the far tails of the distribution. With the chain-rule, however, we can assume a suitable canonical reference hypothesis at the start of each stage, and only need to derive the feature distribution imposed on the output of that stage.

As long as the reference hypothesis used at each stage meets the stated requirements given in Section 2.1, then the chain as a whole will indeed produce the desired MaxEnt projected PDF, which is the PDF with maximum entropy among all PDFs that generate the desired output feature distribution p(z) through the combined transformation [8]!

An example of the application of the chain-rule is the computation of MEL frequency cepstral coefficients (MFCC), commonly used in speech processing. Let us consider a frame of data of length *N*, denoted by x. The processing is broken into the following stages:

1. The first step, denoted by $y=T_{1}\left(x\right)$ is to convert x into N/2+1 magnitude-squared discrete Fourier transform (DFT) bins. Under the standard Gaussian assumption (9), the elements of y are independent and have chi-squared statistics (see Section VI.D.1, pp. 47–48 in [12]).
2. The second step is to sum energy in a set of *K* MEL-spaced band functions. This results in a set of *K* band energies. This can be written using the (N/2−1)×K matrix A as the linear transformation $w=A^{\prime }y.$ This feature transformation is explained in Section 3.2 above so an exponential reference distribution can be assumed for y. Care must be taken that the *K* band functions add to a constant—this insures the energy statistic is “contained in the features”.
3. The next step is to compute the log of the *K* band energies, u=log(w). This is a 1:1 transformation for which PDF projection simplifies to computing the determinant of the transformation’s Jacobian matrix (see Section VI.A, p. 46 in [12]).
4. The last step is the discrete cosine transform (DCT), which can be written as a linear transformation $z=C^{\prime }u$. If some DCT coefficients are discarded, then the transformation must be analyzed as in Section 3.1 above by including the energy statistic $t\left(u\right)=u^{\prime }u.$

This example illustrates that complex feature transformations can be easily analyzed if broken into simple steps. More on the above example can be found in Sections V and VI in [8].

### 4.3. Large-*N* Conditional Distributions and Applications.

When the feature value $z^{*}$ is fixed, then sampling x on the manifold $M(z^{*})$, called UMS, has some interesting interpretations relative to maximum entropy. Let the conditional distribution be written $p(x|z^{*})$. Notice that $p(x|z^{*})$ is not a proper distribution since all the probability mass exists on the manifold $M(z^{*})$ of zero volume. Writing down $p(x|z^{*})$ in closed form or determining its mean is intractable. It is useful, however, to know $p(x|z^{*})$ because, for example, the mean of $p(x|z^{*})$ is a point estimate of x based on $z^{*}$, a type of MaxEnt feature inversion. However, depending on the range of x, as exemplified by the three cases in Section 3.1, Section 3.2 and Section 3.3, $p(x|z^{*})$ can be approximated by a *surrogate distribution* (See p. 2461 in [9]). The surrogate distribution is a proper distribution that (a) has its probability mass concentrated near $M(z^{*})$, (b) has constant value on $M(z^{*})$, and (c) has mean value on the manifold, so $\bar{x}\in M\left(z^{*}\right)$. The surrogate distribution therefore meets the same conditions as $p(x|z^{*})$ but is a proper distribution. The mean of the surrogate distribution is a very close approximation to the mean of $p(x|z^{*})$, which can be called th *centroid* of $M(z^{*})$, but can be computed. In Section 3.1, Section 3.2 and Section 3.3, the surrogate distribution is Gaussian, exponential, and truncated exponential, respectively. These are the MaxEnt distributions under applicable constraints. It was shown, for example, when the range of x is the positive quadrant of R, that the centroid corresponds to the classical Maximum Entropy feature inversion approach for a dimension-reducing linear transformation of intensity data, for example to sharpen images blurred by a point-spread function [9]. The method, however, is more general because it can be adapted to different ranges of x [9].

## 5. Applications

### 5.1. Classification

Assume there are *M* classes and the *M* class hypotheses are $H_{1},H_{2}\ldots H_{M}.$ The general form of the classifier by applying Bayes theorem and (8) is given by

(15) $$\hat{m}=\arg\max_{m}p\left(x|H_{m}\right)\;p\left(H_{m}\right),$$

where $p(H_{m})$ is the prior class probability, and $p(x|H_{m})$ is a PDF estimate for class hypothesis $H_{m}$. For the classification problem, there are many classifier topologies for using (8) to construct $p(x|H_{m})$.

1. Class-specific features. One can specify a different feature transformation per class, $z^{m}=\Phi^{m}\left(x\right)$,
  $$p\left(x|H_{m}\right)=\frac{p_{0}^{*}\left(x\right)}{p_{0}^{*}\left(z^{m}\right)}\;p\left(z^{m}|H_{m}\right),$$
  but the numerator is common, so the classifier rule becomes
  $$\hat{m}=\arg\max_{m}\frac{p(z^{m}|H_{m})}{p_{0}^{*}\left(z^{m}\right)}.$$
  This amounts to just comparing the likelihood ratio between class hypothesis $H_{m}$ and the reference distribution, computed using a class-dependent feature [16].
2. It is not necessary to use a common reference hypothesis. A class-dependent reference hypothesis can be selected so that the feature is an approximately sufficient statistic to discriminate the given class from the class-dependent reference hypothesis. Then,
  $$p\left(x|H_{m}\right)=\frac{p_{0,m}\left(x\right)}{p_{0,m}\left(z^{m}\right)}\;p\left(z^{m}|H_{m}\right),$$
  where $p_{0,m}\left(x\right)$ is the class-dependent reference hypothesis. Note that, when using the chain-rule (14), there is not a single reference hypothesis associated with each class, but a series of stage-wise reference hypotheses. Note that here we have relaxed the MaxEnt requirement for the reference hypothesis.
3. Using a different feature to test each class hypothesis is not always a good idea. Some data can be “contaminated” with noise or interference, so they may not be suitable to test a hypothesis with just one feature. In this case, a *class-specific feature mixture* (CSFM) [17,18,19] may be appropriate. For the CSFM, we define a set of feature transformations $\left\{\Phi^{1}\left(x\right),\Phi^{2}\left(x\right),\ldots \Phi^{M}\left(x\right)\right\}$. (We assume here that the number of feature transformations equals the number of classes, but this is not necessary.) Then, $p(x|H_{m})$ is constructed as a mixture density using all the features:
  $$p\left(x|H_{m}\right)=\sum_{l=1}^{M}w_{m,l}\;\frac{p_{0,l}^{*}\left(x\right)}{p_{0,l}^{*}\left(z^{l}\right)}\;p\left(z^{l}|H_{m}\right),$$
  where $p_{0,l}^{*}\left(x\right)$ is the MaxEnt reference hypothesis corresponding to each feature transformation $\Phi^{l}\left(x\right)$.
4. To solve the classification problem (15), it is necessary to obtain a segment of data x that can be classified into one of *M* classes. The problem is often not that simple, and the location of the classifiable “event” may be unknown within a longer data recording, or the data recording may contain multiple events from multiple classes. Using MaxEnt PDF projection, it is possible to solve the data segmentation problem simultaneously with the classification problem [20,21].

### 5.2. Other Applications

MaxEnt PDF projection has applications in the analysis of networks and feature transformations. For example in neural networks, it is possible to view a feed-forward neural network as a generative network, a duality relationship between two opposing types of networks [22]. In addition, the restricted Boltzmann machine (RBM) can be used as a PDF estimator with tractable distribution [13]. In feature inversion, MaxEnt PDF projection can be used to find MaxEnt point-estimates of the input data x based on fixed values of the feature [9].

## 6. Conclusions

In this short paper, the method of maximum entropy PDF projection was presented as a generalization of Jaynes’ maximum entropy principle with moment constraints. The mathematical basis of maximum entropy PDF projection was reviewed and practical considerations and applications were presented.
